# Exploring near-infrared autofluorescence properties in parathyroid tissue: an analysis of fresh and paraffin-embedded thyroidectomy specimens

**DOI:** 10.1117/1.JBO.30.S1.S13702

**Published:** 2024-07-18

**Authors:** Bo Wang, Chi-Peng Zhou, Wei Ao, Shao-Jun Cai, Zhi-Wen Ge, Jun Wang, Wen-Yu Huang, Jia-Fan Yu, Si-Bin Wu, Shou-Yi Yan, Li-Yong Zhang, Si-Si Wang, Zhi-hong Wang, Surong Hua, Amr H. Abdelhamid Ahmed, Gregory W. Randolph, Wen-Xin Zhao

**Affiliations:** a Fujian Medical University Union Hospital, Department of Thyroid Surgery, Fuzhou, China; b Fujian Clinical Research Center for Precision Management of Thyroid Cancers, Fuzhou, China; c Harvard Medical School, Division of Thyroid and Parathyroid Endocrine Surgery, Department of Otolaryngology-Head and Neck Surgery, Massachusetts Eye and Ear Infirmary, Boston, Massachusetts, United States; d Fujian Medical University Union Hospital, Department of Pathology, Fuzhou, China; e The First Affiliated Hospital of China Medical University, Department of Thyroid Surgery, Shenyang, China; f Peking Union Medical College, Department of General Surgery, Peking, China; g Massachusetts General Hospital, Harvard Medical School, Department of Surgery, Boston, Massachusetts, United States

**Keywords:** near-infrared autofluorescence, parathyroid gland, thyroidectomy, central neck

## Abstract

**Significance:**

Near-infrared autofluorescence (NIRAF) utilizes the natural autofluorescence of parathyroid glands (PGs) to improve their identification during thyroid surgeries, reducing the risk of inadvertent removal and subsequent complications such as hypoparathyroidism. This study evaluates NIRAF’s effectiveness in real-world surgical settings, highlighting its potential to enhance surgical outcomes and patient safety.

**Aim:**

We evaluate the effectiveness of NIRAF in detecting PGs during thyroidectomy and central neck dissection and investigate autofluorescence characteristics in both fresh and paraffin-embedded tissues.

**Approach:**

We included 101 patients diagnosed with papillary thyroid cancer who underwent surgeries in 2022 and 2023. We assessed NIRAF’s ability to locate PGs, confirmed via parathyroid hormone assays, and involved both junior and senior surgeons. We measured the accuracy, speed, and agreement levels of each method and analyzed autofluorescence persistence and variation over 10 years, alongside the expression of calcium-sensing receptor (CaSR) and vitamin D.

**Results:**

NIRAF demonstrated a sensitivity of 89.5% and a negative predictive value of 89.1%. However, its specificity and positive predictive value (PPV) were 61.2% and 62.3%, respectively, which are considered lower. The kappa statistic indicated moderate to substantial agreement (kappa = 0.478; P<0.001). Senior surgeons achieved high specificity (86.2%) and PPV (85.3%), with substantial agreement (kappa = 0.847; P<0.001). In contrast, junior surgeons displayed the lowest kappa statistic among the groups, indicating minimal agreement (kappa = 0.381; P<0.001). Common errors in NIRAF included interference from brown fat and eschar. In addition, paraffin-embedded samples retained stable autofluorescence over 10 years, showing no significant correlation with CaSR and vitamin D levels.

**Conclusions:**

NIRAF is useful for PG identification in thyroid and neck surgeries, enhancing efficiency and reducing inadvertent PG removals. The stability of autofluorescence in paraffin samples suggests its long-term viability, with false positives providing insights for further improvements in NIRAF technology.

## 1 Introduction

Parathyroid glands (PGs) are small endocrine organs that regulate calcium homeostasis by secreting parathyroid hormone (PTH) in response to changes in serum calcium levels. PGs are usually located on the posterior surface of the thyroid gland, but their position and number can vary widely. The normal number of PGs is four, but some people may have three or more and occasionally up to eight or more.^1^ Identifying and preserving PGs during thyroid surgery is crucial to prevent postoperative hypoparathyroidism, which can cause hypocalcemia, tetany, and other complications.^2^

PGs, typically located on the posterior surface of the thyroid, measuring ∼3 to 5 mm and resembling fatty tissue, are particularly vulnerable during thyroid surgery. Identifying PGs during thyroid surgery can be challenging, especially for inexperienced surgeons or in cases of anatomical variations.^3^ Several methods have been proposed to assist in PG identification, such as intraoperative PTH test assays, frozen section analysis,^4^ optical imaging techniques,^5^ and near-infrared autofluorescence (NIRAF).^6^ These methods have limitations in terms of accuracy, cost, availability, and invasiveness.^7^

NIRAF is a novel technique that exploits the natural autofluorescence of PGs under near-infrared light. It has been shown to be effective and safe for identifying PGs during thyroid surgery in several studies.^8^ NIRAF can improve the identification and preservation of PGs during thyroid surgery and reduce the risk of postoperative hypoparathyroidism.^6^^,^^9^^,^^10^ A contemporary international study led by the American Head and Neck Society (AHNS) reviewed PG identification and vascular assessment using different NIRAF modalities (both label-free and indocyanine green-based) and compared the pros and cons of different techniques using probe-based and camera-based NIRAF devices in addition to a manual of use.^7^ However, these studies were based on the prior judgment of suspicious tissues by experienced surgeons, overlooking potential issues with false positives.^11^

Therefore, the aim of this study is to assess the performance of NIRAF for identifying PGs in postoperative specimens. We compared NIRAF to both junior and senior surgeon visual inspections. We also analyzed the distribution of PGs on the thyroid map and the sources of false positives for NIRAF. We hypothesized that NIRAF would exhibit higher sensitivity in elucidating the identity of PGs, albeit with lower specificity and positive predictive value (PPV) due to false-positive results. A thorough analysis of false-positive issues could provide new insights for subsequent artificial intelligence-based NIRAF identification approaches.

## 2 Methods

### 2.1 Clinical Study Design

We conducted a prospective study of 101 patients who underwent thyroidectomy with or without central lymph node dissection for thyroid cancer at a tertiary referral center between November 2022 and January 2023. The study followed the Strengthening the Reporting of Observational Studies in Epidemiology reporting guidelines. The study was approved by the Institutional Review Board, and informed consent was obtained from all patients.

### 2.2 Setting

The study was conducted at the Department of Thyroid Surgery of our institution, which is a high-volume center for thyroid surgery.

### 2.3 Participants

The participants were patients with papillary thyroid carcinoma who underwent thyroid surgery at our institution. The inclusion criteria were as follows: age 18 years or older, diagnosis of papillary thyroid carcinoma confirmed by fine-needle aspiration biopsy, and indication for thyroidectomy with central lymph node dissection. The exclusion criteria were as follows: previous history of thyroid or parathyroid surgery, previous history of radiation exposure to the neck, and previous history of hyperparathyroidism or hypoparathyroidism. All patients provided written informed consent before participating in the study.

### 2.4 Interventions

All surgeries were performed by experienced endocrine surgeons using conventional techniques or endoscopic techniques. The identification and preservation of PGs were based on visual inspection, tactile sensation, and blood supply. The PGs were classified into three types according to their location relative to the thyroid capsule: type A1 (extrathyroidal PGs), type A2 (partially exposed PGs), and type A3 (intrathyroidal PGs). The PGs were either preserved *in situ* or transplanted into the left forearm muscle if they were devascularized or inadvertently removed.

After surgery, all specimens were examined by three different methods in order: NIRAF, senior surgeon examination, and junior surgeon examination. NIRAF was performed using a commercially available NIRAF device (ARGOS NIR-300PT, Microscopic Intelligence Co., Hunan, China) that emits near-infrared light at 785 nm and detects autofluorescence at 820 to 850 nm. The senior surgeon examination was performed by a senior endocrine surgeon who had more than 5 years of experience in endocrine surgery. The junior surgeon examination was performed by a first-year resident who had limited experience in endocrine surgery.

### 2.5 Main Outcomes and Measures

The main outcomes and measures were as follows:

The number, location, and type of PGs were recorded for each method, and fluorescence intensity was recorded for the NIRAF method. NIRAF suspicious positives were identified in NIRAF images as uniformly high-intensity white circular or elliptical-like shapes, with signals at least twice as intense as the surrounding background fluorescence.

**Fig. 1:**
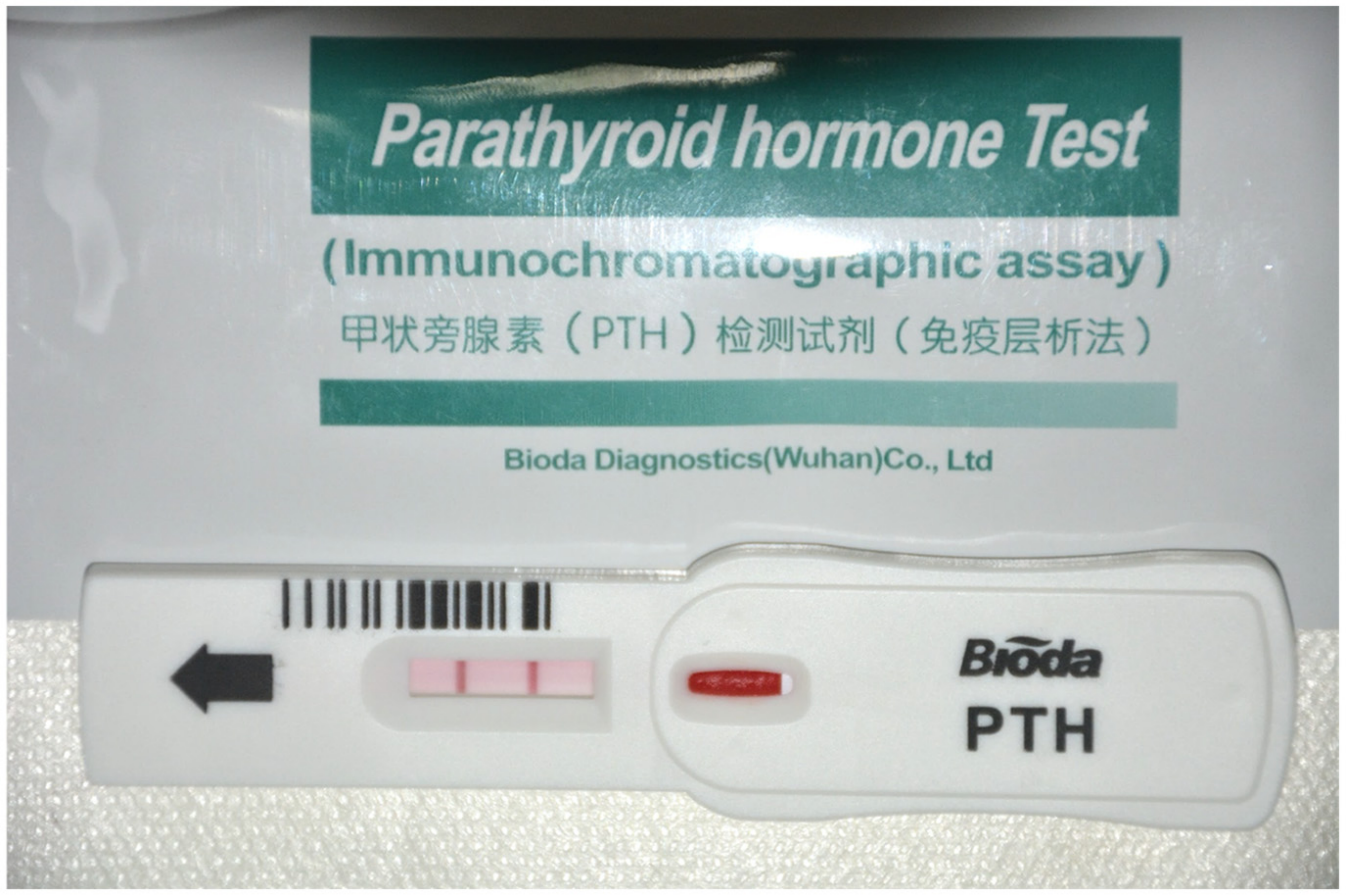
Confirmation of PG tissue by PTH test assay.

The presence or absence of PG tissue was confirmed using PTH test assays (Bioda Diagnostics Co., Wuhan, China) (Fig. 1) or pathological assessment. A video of how to use this PTH test assay is available in our previous paper.^12^

The examination time was defined as the time from the start to the end of each PG assessment method.

The performance of each method was evaluated using the following parameters: examination time, identification rate, true positive rate, false-positive rate, sensitivity, specificity, PPV, negative predictive value (NPV), accuracy, and kappa coefficient.

The distribution of PGs on the thyroid map was analyzed according to their location in the postoperative specimens.

The sources of error for NIRAF were analyzed according to the type and frequency of false-positive and false-negative results.

## 3 Pathological Study

Fifty thyroid specimens containing PGs, collected between 2011 and 2021, were analyzed for their fluorescence characteristics. The central and maximum fluorescence of the parathyroid tissues were quantified using ImageJ software, and these values were compared with the background fluorescence of the paraffin-embedded tissue. Immunohistochemistry was performed on these specimens to detect calcium-sensing receptor (CaSR) and vitamin D. The intensity and proportion of positive fluorescence were analyzed to further explore the correlation between fluorescence intensity and protein expression. The mean and central fluorescence intensities of PT, as well as background fluorescence intensity, were calculated using ImageJ software. Both mean and central fluorescence intensities are normalized against the background fluorescence.

### 3.1 Statistical Analysis

Statistical analysis was performed using SPSS software version 26 (IBM Corp., Armonk, New York, United States). Descriptive statistics were used to summarize the data. Continuous variables were expressed as the mean ± standard deviation (SD) and compared using the Student’s t-test or one-way analysis of variance (ANOVA). Categorical variables were expressed as frequencies and percentages and compared using the chi-square test or Fisher’s exact test. A P value <0.05 was considered statistically significant.

### 3.2 Ethical Approval

This study was conducted in accordance with the principles of the Declaration of Helsinki and approved by the Institutional Review Board of our institution (2023KY053). All patients provided written informed consent before participating in the study.

### 3.3 Trial Registration

This study was registered at ClinicalTrials.gov with the identifier NCT05684029.

### 3.4 Data Sharing Statement

The data that support the findings of this study are available from the corresponding author upon reasonable request.

## 4 Results

### 4.1 Patient Demographic and Clinical Characteristics

**Fig. 2:**
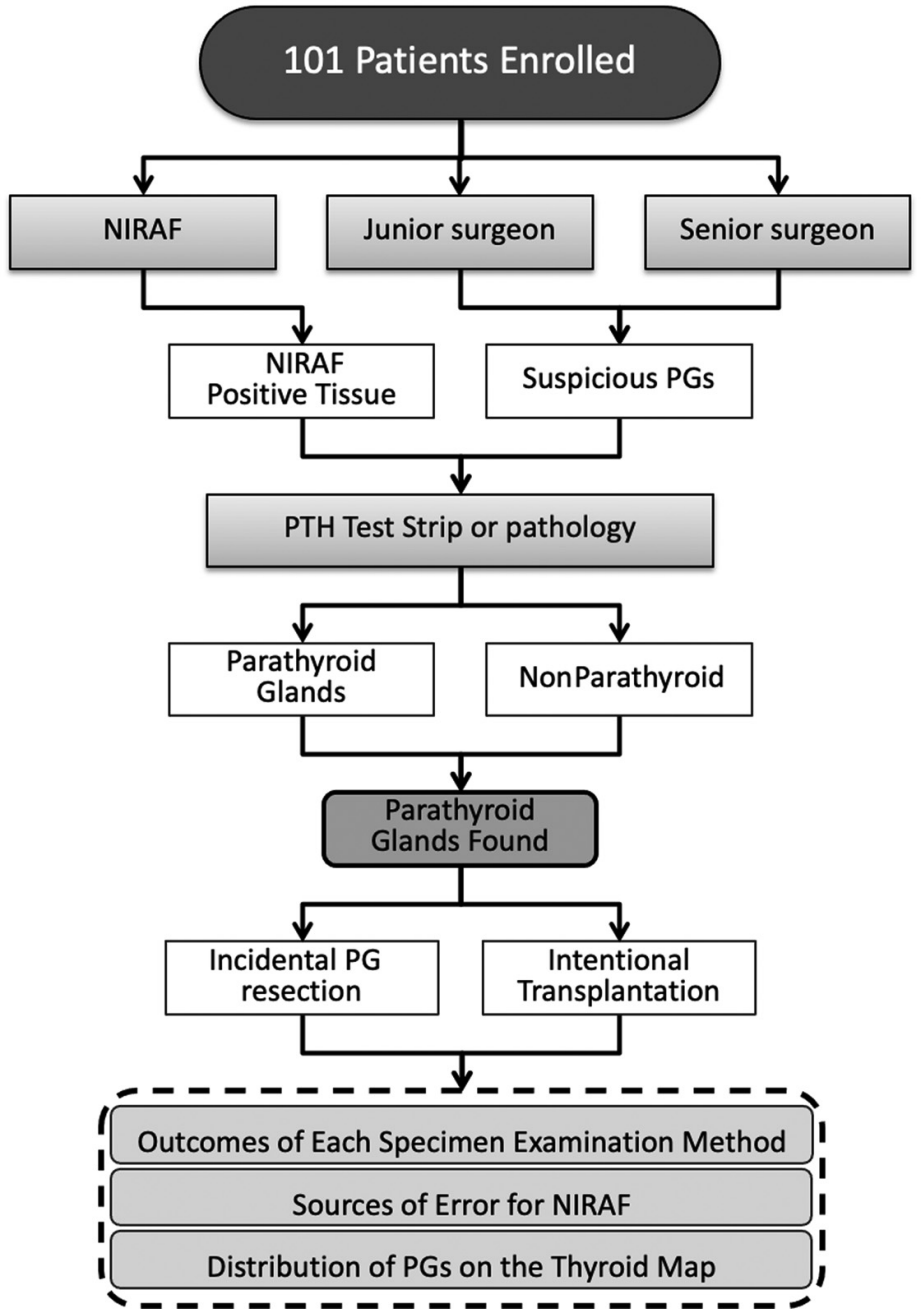
Workflow integration of NIRAF in surgical specimen examination.

A total of 101 patients with papillary thyroid carcinoma who underwent thyroidectomy with central lymph node dissection were included in this study. The flowchart of this study is displayed in Fig. 2. The demographic and clinical characteristics of the participants are summarized in Table 1. The mean age of the patients was 41.3 years (SD, 11.7 years), and 75.2% were female. The mean body mass index was $22.99\;\mathrm{kg}/m^{2}$ (SD, $3.4\;\mathrm{kg}/m^{2}$). All patients had a pathologic diagnosis of papillary thyroid carcinoma, and 21.8% had coexisting Hashimoto’s thyroiditis. The majority of patients (65.3%) underwent lobectomy with central lymph node dissection, followed by total thyroidectomy with central lymph node dissection (23.8%) and bilateral subtotal thyroidectomy (two cases) or lobectomy plus contralateral subtotal thyroidectomy (nine cases) with central lymph node dissection (10.9%) because of isthmic thyroid cancer. The mean PTH level 1 day post-operation was 1.72  pmol/L (SD, 1.09  pmol/L), and the mean serum calcium level 1 day post-operation was 2.28  mmol/L (SD, 0.12  mmol/L). In addition, 50 pathological specimens were from paraffin-embedded tissues of accidentally excised PGs during thyroid surgeries over the past decade. All patients were diagnosed with thyroid cancer and underwent central lymph node dissection.

**Table 1:** Demographic and clinical characteristics of the participants.

Variable	Value
Age, mean (SD), y	41.3 (11.7)
Sex, no. (%)	
Female	76 (75.2)
Male	25 (24.8)
BMI, mean (SD), $\mathrm{kg}/m^{2}$	22.99 (3.4)
Pathologic diagnosis, no. (%)	
Papillary thyroid carcinoma	101 (100.0)
Coexisting Hashimoto’s thyroiditis	22 (21.8)
Type of surgery, no. (%)	
Total thyroidectomy with CLND	23 (22.8)
Lobectomy with CLND	67 (66.3)
Subtotal with CLND^a^	11 (10.9)
Total number of PGs identified	105
PTH level day 1 post-operation mean (SD), pmol/L^b^	1.72 (1.09)
Serum calcium level day 1 post-operation mean (SD), mmol/L	2.28 (0.12)

a Include two cases of bilateral subtotal thyroidectomy and nine cases of lobectomy with contralateral subtotal thyroidectomy isthmic thyroid cancer because of isthmus thyroid cancer.

b PTH normal range 1.3−9.3  pmol/L.

Note: CLND, central lymph node dissection; SD, standard deviation; y, year; cm, centimeter.

### 4.2 Outcomes of NIRAF Versus Visual Identification

**Fig. 3:**
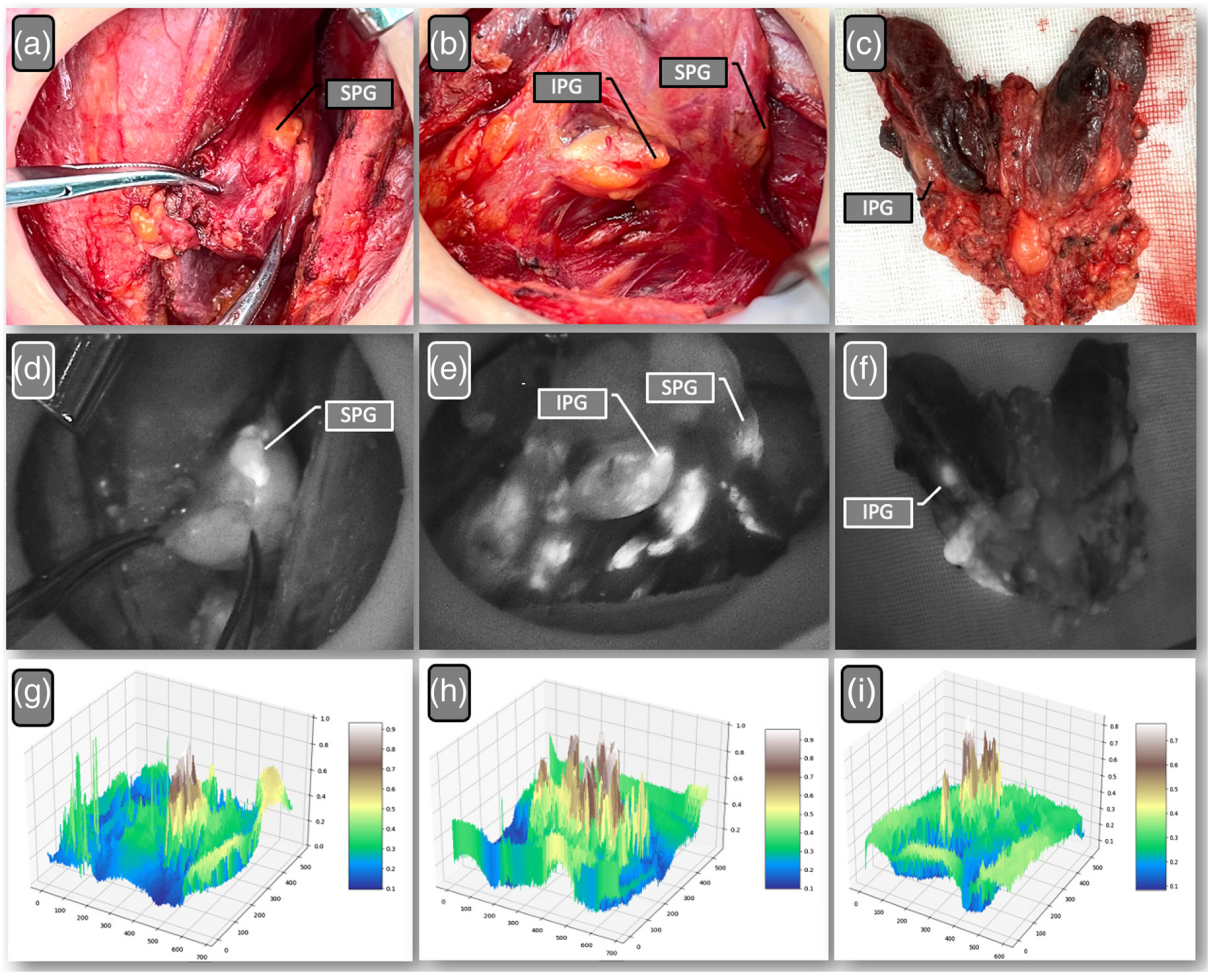
Images of PGs detected by visual inspection and NIRAF with corresponding topographic maps. Panels (a)–(c) depict the PGs as seen in the surgical field, typically measuring 3 to 5 mm and resembling yellow fatty tissue. Specifically, panel (a) shows a superior PG without significant false positives, panel (b) illustrates both superior and inferior PGs with numerous false positives, and panel (c) displays PGs included within the specimen. Panels (d)–(f) present the corresponding near-infrared views, where the PGs emit a white signal, highlighting their presence. Panels (g)–(i) showcase the corresponding grayscale topographic maps, which provide a clear indication of a good signal-to-noise ratio, aiding in the effective examination of PGs in the specimens. SPT, superior parathyroid; IPT, inferior parathyroid.

The performance metrics and kappa coefficients for identifying PGs in postoperative specimens are detailed in Table 2. The NIRAF method exhibited the shortest examination time, averaging 11.8 s (SD = 4.9), compared with the senior surgeon at 122.6 s (SD = 43.8) and the junior surgeon at 162.7 s (SD = 48.1). NIRAF achieved the highest counts of positive identification (151 of 252 cases or 59.9%), sensitivity (94 of 105 cases or 89.5%), and NPV (90 of 101 cases or 89.1%). However, it showed the lowest specificity (90 of 147 cases or 61.2%), PPV (94 of 151 cases or 62.3%), and kappa coefficient (0.478; P<0.001). The senior surgeon demonstrated the highest specificity (100 of 116 cases or 86.2%), PPV (104 of 122 cases or 85.3%), and kappa coefficient (0.847; P<0.001), indicating almost perfect agreement with the reference standard (PTH test assay or pathological assessment). The junior surgeon had the lowest kappa coefficient (0.381; P<0.001), signaling moderate agreement with the reference standard. These findings underscore the utility of NIRAF in reducing the risk of accidental PG removal. Figure 3 presents images of PGs identified by both visual inspection and NIRAF during and post-surgery.

**Table 2:** Performance and kappa coefficient of each examination method for identifying PGs in postoperative specimens.

Variable	NIRAF	Junior surgeon visual identification	Senior surgeon visual identification
Examination time, mean (SD), s	11.8 (4.9)	162.7 (48.1)	122.6 (43.8)
Positive number, no./total no. (%)	151/252 (59.9)	115/216 (53.2)	120/223 (53.8)
True positive rate, no./total no. (%)	94/105 (89.5)	76/104 (73.1)	104/105 (99.0)
False-positive rate, no./total no. (%)	57/147 (38.8)	39/112 (34.8)	16/116 (13.8)
Incidental PG resection rate, no./total no. (%)^a^	11/105 (10.5)	29/105 (27.6)	1/105 (1.0)
Sensitivity, no./total no. (%)	94/105 (89.5)	76/105 (73.1)	104/105 (99.0)
Specificity, no./total no. (%)	90/147 (61.2)	73/112 (65.2)	100/116 (86.2)
Overall concordance rate (%)	184/252 (73.0%)	149/216 (69.0%)	204/223 (91.5%)
Positive predictive value, no./total no. (%)	94/151 (62.3)	76/115 (66.1)	104/122 (85.3)
Negative predictive value, no./total no. (%)	90/101 (89.1)	73/101 (72.3)	100/101 (99.0)
Kappa coefficient	0.478 (P<.001)	0.381 (P<0.001)	0.847 (P<0.001)

Note: The kappa coefficient was calculated as a measure of agreement between each method and the reference standard (PTH test assay or pathological assessment).

a Intentional parathyroid autotransplantation is the decision to transplant normal parathyroid tissue during thyroid surgery to prevent permanent hypoparathyroidism when there is no anatomical possibility for preservation of the PG *in situ* or when there is devascularization of the gland.^13^ Data well no include in the incidental PG resection rate.

NIRAF, near-infrared autofluorescence; SD, standard deviation; s, second; PTH, parathyroid hormone; PGs, parathyroid glands.

### 4.3 Stability of Autofluorescence

**Fig. 4:**
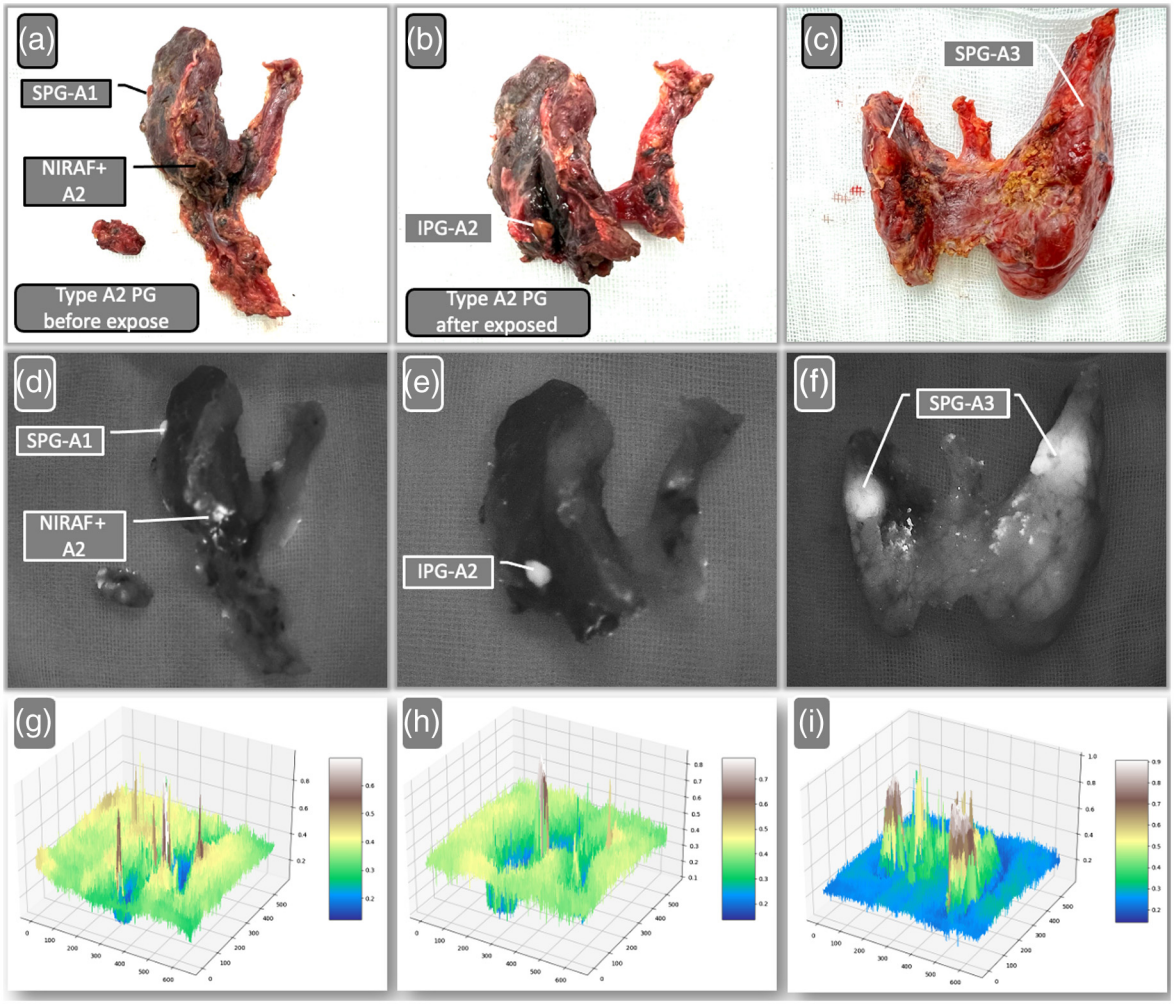
Examples of tissue penetration capabilities of PGs by visual inspection and NIRAF examination of specimens. (a), (b) NIRAF images of the frontal and lateral PGs, respectively. (c), (d) Type A2 PGs after exposure. (e), (f) Bilateral type A3 PGs (parathyroid adenomas). (b), (f) Tissue penetration of NIRAF. The arrows indicate PGs. SPT, superior parathyroid; IPT, inferior parathyroid.

The examination of additional paraffin-embedded specimens collected over nearly a decade demonstrates that the autofluorescence of PG tissues remains stable, even after being preserved in paraffin for up to 10 years. As shown in Fig. 4 and Table 3, there are no indications of diminished fluorescence intensity over the years. Nonetheless, a definitive correlation between autofluorescence and the expression levels of CaSR and vitamin D has yet to be confirmed.

**Table 3:** Summary of fluorescence intensity analysis across different years.

Year	Case	Mean PT fluorescence intensity^a^	Center PT fluorescence intensity^b^	CaSR score	Vitamin D score
		Mean ± SD	Mean ± SD	Mean ± SD	Mean ± SD
2011	6	1.575 ± 0.124	1.697 ± 0.156	9.0 ± 0.0	5.33 ± 2.50
2013	7	1.509 ± 0.226	1.710 ± 0.264	9.0 ± 0.0	4.43 ± 1.62
2016	8	1.360 ± 0.160	1.513 ± 0.182	9.0 ± 0.0	4.63 ± 2.62
2018	7	1.371 ± 0.235	1.533 ± 0.265	9.0 ± 0.0	5.14 ± 1.46
2019	8	1.406 ± 0.165	1.620 ± 0.163	9.0 ± 0.0	5.88 ± 1.55
2020	6	1.448 ± 0.238	1.638 ± 0.287	9.0 ± 0.0	6.33 ± 2.58
2021	8	1.405 ± 0.148	1.588 ± 0.194	9.0 ± 0.0	6.38 ± 2.97
P value	—	0.348	0.540	NA	0.530

Notes (a) and (b): The mean and central fluorescence intensities of parathyroid tissue, along with the background fluorescence intensity of the paraffin, were determined using ImageJ software. Both the mean and central fluorescence intensities have been normalized to the background fluorescence. No significant differences were found in the mean or central fluorescence intensities or in the vitamin D score over the years, as per the analysis conducted using ANOVA followed by an least significant difference post hoc test. NA indicates that the CaSR score could not be calculated due to zero variance.

### 4.4 NIRAF’s Penetration Capabilities and Sources of Errors

**Fig. 5:**
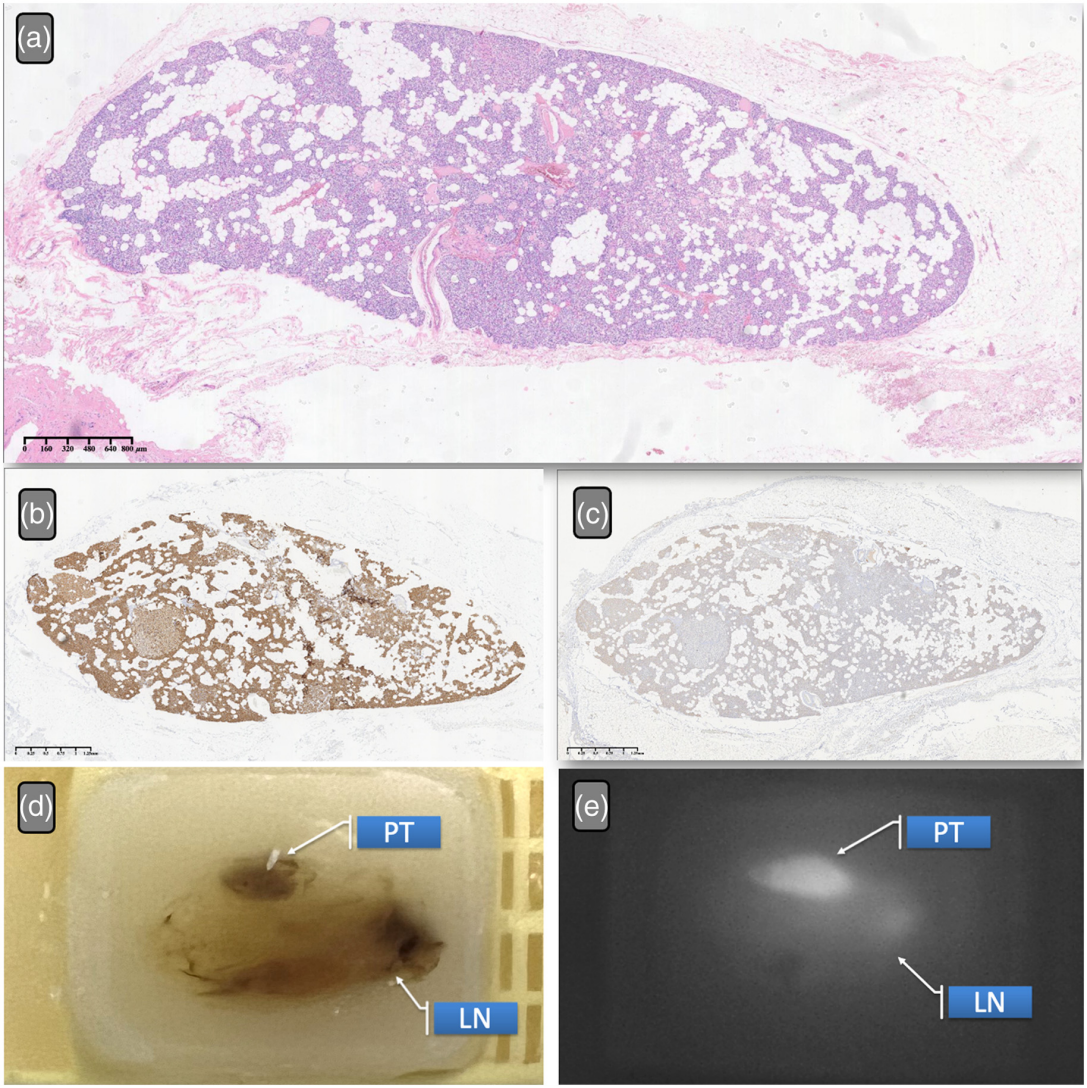
Immunohistochemistry and NIRAF of the parathyroid gland in paraffin specimens. Panel (a) displays the PG with hematoxylin and eosin (HE) staining, shown in blue; panel (b) represents the results of CaSR immunohistochemistry; panel (c) depicts vitamin D immunohistochemistry; panel (d) shows a paraffin-embedded specimen of the PG (indicated by the arrow as PG) from the year 2011; panel (e) illustrates the near-infrared autofluorescence of the paraffin-embedded specimen. The lymph nodes also display a small amount of positive signal (indicated by the arrow as LN).

NIRAF demonstrated excellent penetration capabilities, particularly effective in identifying PGs that were partially exposed (type A2) or embedded within the thyroid gland (type A3), according to the Chinese classification. This effectiveness was shown in five specific cases, underscoring NIRAF’s utility in challenging anatomical scenarios. Table 4 also outlines the sources of errors for NIRAF. The primary sources of false positives included brown fat, constituting 86.0% (49 out of 57) of cases, and eschar at 12.3% (7 out of 57). A colloidal nodule was a minor source, accounting for only 1.7% (1 out of 57), with no errors reported in lymph nodes. The most frequent issue of false negatives was due to the absence of detectable fluorescence on the surface of PGs, observed in 11 cases (Fig. 5).

**Table 4:** Sources of error for NIRAF.

Type and source of error	Number of cases	Percentage (%)
False-positive results	—	—
Brown fat	49	86.0
Eschar	7	12.3
Colloidal nodule	1	1.7
Lymph node	0	0
Total false-positive results	57	—
False-negative results	—	—
Lack of fluorescence on the surface	11	100.0
Total false-negative results	11	—
Penetration abilities	—	—
Type A2 and A3 PGs^a^	5	100.0

Note: False-positive results were defined as non-PGs that were mistakenly identified as PGs by NIRAF. False-negative results were defined as PGs that were missed by NIRAF.

a According to the PG classification in China, type A2 (partially exposed PGs) and A3 (intrathyroidal PGs).

Note: NIRAF, near-infrared autofluorescence; PGs, parathyroid glands.

### 4.5 Distribution of Inadvertently Excised PGs on the Thyroid Map

Table 5 illustrates the distribution of PGs on the thyroid map. The most frequent location for these glands was the anterior surface of the thyroid gland, where 85.7% (90 of 105) were found, followed by the inferior pole with 57.1% (60 of 105), central compartment lymph nodes at 23.8% (25 of 105), the superior pole at 19.0% (20 of 105), and the posterior surface at 14.3% (15 of 105). No PGs were located in the isthmus or mediastinum.

**Table 5:** Distribution of PGs on the thyroid map.

Location	Number of PGs	Percentage (%)
Anterior surface	90	85.7
Posterior surface	15	14.3
Superior pole (L1 and R1)	20	19.0
Inferior pole	60	57.1
Isthmus	0	0
Central compartment lymph nodes	25	23.8
Mediastinum	0	0
Total	105	100

Note: PGs, parathyroid glands.

## 5 Discussion

This prospective study evaluated the performance of NIRAF, senior surgeons, and junior surgeons in identifying PGs in postoperative specimens of patients with papillary thyroid carcinoma who underwent thyroidectomy with central lymph node dissection. The main findings were that NIRAF had the highest sensitivity and NPV but the lowest specificity and PPV among the three methods. The senior surgeon had the best agreement with the reference standard, followed by the junior surgeon. The NIRAF method had poor agreement with the reference standard mainly due to the high rate of false-positive results caused by brown fat, eschar, and colloidal nodules. The false-negative results of NIRAF were mainly due to the lack of fluorescence on the surface of PGs. However, NIRAF demonstrated an excellent penetration ability to detect partially exposed or intrathyroidal PGs.

NIRAF is a novel technique that utilizes the autofluorescence property of PGs under near-infrared light. Previous studies have shown that NIRAF can facilitate intraoperative identification and preservation of PGs during thyroid surgery.^14^^–^^18^ However, few studies have evaluated the accuracy of NIRAF in identifying PGs in postoperative specimens. In this study, we found that NIRAF had a high sensitivity (89.5%) and NPV (89.1%) in detecting PGs in postoperative specimens, which means that NIRAF can effectively identify most of the PGs and reduce the risk of missing them. However, NIRAF also had a low specificity (61.2%) and PPV (62.3%) in identifying PGs in postoperative specimens, which means that NIRAF can mistakenly identify many nonparathyroid tissues as PGs and increase the risk of false-positive results. This result, contradicting the previous studies,^14^^–^^17^ may be attributed to the fact that most of the previous studies limited themselves to intraoperative use and performed the visual assessment of senior surgeons, which objectively improved the specificity of NIRAF. Therefore, NIRAF alone may not be sufficient to confirm the presence or absence of PGs in postoperative specimens, and additional methods such as PTH test assays or pathological assessments are needed to verify the results. In addition, compared with angiography with indocyanine green fluorescence, NIRAF cannot distinguish the vessel condition of PG.^19^

Previous studies have speculated that the autofluorescence of PGs is due to the expression of CaSR or vitamin D within the glands.^20^^–^^22^ Through a retrospective analysis of parathyroid pathological specimens over nearly a decade, we found that the intensity of parathyroid autofluorescence does not change over time, and false positives associated with autofluorescence also occur. Immunohistochemical analysis revealed that the results are insufficient to support a correlation between CaSR and vitamin D expression and fluorescence intensity. The primary sources of error for NIRAF were brown fat, eschar, colloidal nodules, and lack of fluorescence on the surface of PGs. Brown fat, accounting for 86.0% of the false positives, is a type of adipose tissue rich in mitochondria, similar to parathyroid tissues. Brown fat can emit autofluorescence under near-infrared light due to its high content of flavoproteins and cytochromes in mitochondria.^23^^,^^24^ Eschar, formed as a scab or piece of dead tissue after cauterization or electrocoagulation, can also emit autofluorescence due to its high content of porphyrins and other organic compounds. Colloidal nodules, benign thyroid nodules containing colloid material within follicular cells, can emit autofluorescence due to their high content of thyroglobulin and other proteins.^25^ These tissues can mimic the autofluorescence of PGs due to their similar biochemical components, such as reduced nicotinamide adenine dinucleotide, flavin adenine dinucleotide, tryptophan, tyrosine, and phenylalanine. However, the spectral characteristics of these proteins or fluorescent groups do not match the wavelength of parathyroid autofluorescence, suggesting that parathyroid autofluorescence may result from a combination of various proteins.

Lack of fluorescence on the surface of PGs can cause false-negative results by NIRAF. This may be due to several factors, such as ischemia or necrosis of PGs during surgery or specimen handling. The hemoglobin in this process absorbs most of the near-infrared light. Type A2 and A3 PGs according to the Chinese classification are those that are partially or completely inside the thyroid gland. These types of PGs are more difficult to identify by NIRAF because they are covered by thyroid tissue and have less exposure to near-infrared light. Nevertheless, in this study, we found two partially exposed PGs and two intraglandular parathyroid adenomas that were not identified in subsequent examinations by senior and junior physicians. This demonstrates the excellent penetration ability of NIRAF. We estimate that the depth of NIRAF is 1 to 3 mm and is related to the volume of the PG. This result is consistent with the literature report^18^ and also dispels concerns that the camera system is affected by ambient light, tissue depth, and other factors.^7^

The senior surgeon demonstrated superior capability in identifying postoperative PGs compared with other methods, potentially attributable to their extensive experience, in-depth knowledge, adept techniques, and intricate familiarity with the parathyroid anatomy. Their method, aligning closely with the reference standard (kappa coefficient = 0.847), underscores senior surgeon’s high reliability. Nonetheless, this approach demands a vast accumulation of experience and extended inspection durations, making it less feasible in terms of cost and widespread adoption. Our study found the overall concordance rate of the camera-based NIRAF detection to be at 73%, a stark contrast to the 92.7% accuracy rate of the probe-based PTeye® system documented in the literature.^26^^–^^28^ This discrepancy suggests that junior surgeons using a probe-based system may inadvertently identify other tissues, such as brown fat, as parathyroids. As a result, our research serves as an essential complement, highlighting the need for standardized practices of near-infrared parathyroid autologous fluorescence technology. Although this technology holds promise in swiftly identifying suspicious parathyroids and potentially shortening surgical times, an over-reliance on it, especially by less-experienced doctors, may result in misidentifications. To bolster accuracy, it is advisable to complement this method with rapid tissue fluid PTH assays or pathological examinations. Furthermore, in-depth training on NIRAF and adherence to a standardized protocol are critical to mitigating risks associated with unintended parathyroid removals.^18^^,^^29^^,^^30^

Considering the distribution of inadvertently removed thyroid tissues,^31^ we recommend focusing the NIRAF scanning and manual examination by surgeons on the ventral side of the thyroid, the area between the inferior thyroid artery and the central compartment, and the upper and lower borders of the superior pole near Zuckerkandl’s tubercle (see Table 5). Due to NIRAF’s high sensitivity and quick scanning capabilities, it is beneficial to initially target these areas when searching for PGs. In addition, to confirm the presence of parathyroid tissue in questionable specimens, we advise using a PTH test or a pathological assessment (see Fig. 1), thus preventing the transplantation of tissues mistakenly identified as PGs. If these approaches do not locate PGs, we suggest dissecting the thyroid tissue along its largest cross-section and through the Zuckerkandl’s tubercle to search for potentially exposed or intrathyroidal PGs.

This study demonstrates the practical utility of NIRAF as an effective method for identifying PGs in postoperative specimens and verifying their condition post-surgery. Its application significantly eases the concerns of surgeons and patients about the status of PGs after surgery.^29^^,^^32^ Economically, NIRAF offers considerable advantages over other technologies. Unlike probe-based systems such as PTeye®,^10^ which rely on disposable optical fiber probes, camera-based NIRAF systems necessitate only a one-time investment in equipment and do not require specialized consumables. This not only reduces the overall cost for patients but also facilitates easier clinical adoption. The potential for developing specialized optical instruments tailored for identifying PGs, such as open-configured optical microscopes and portable parathyroid detection devices, which have not yet been reported, further underscores the innovative prospects of NIRAF. While the efficacy of NIRAF is well documented, additional research is essential to enhance the technology. Currently, NIRAF faces issues with false positives and significant variability across different devices, presenting ongoing challenges for further developments in machine learning and artificial intelligence recognition.

## 6 Conclusions

NIRAF has proven to be an invaluable tool for identifying PGs in surgical specimens, seamlessly integrating into the specimen examination phase without interrupting the ongoing surgical procedure. This procedure not only significantly shortens the time required to locate PGs but also reduces the likelihood of their accidental removal. NIRAF’s ability to penetrate tissue facilitates the detection of parathyroid hyperplasia, whether it is fully or partially embedded within the thyroid, offering a distinct advantage in the analysis of surgical specimens. Research on parathyroid specimens preserved in paraffin for up to 10 years has verified that their autofluorescence remains stable, showing no signs of deterioration over time. However, establishing a definitive correlation between this autofluorescence and the expression levels of CaSR and vitamin D, as determined through immunohistochemistry, has yet to be achieved. Due to the frequent occurrence of false positives in both fresh and paraffin-embedded specimens, there is an urgent need to refine the NIRAF imaging algorithm. Enhancing this technology is crucial for reducing false positives, thereby improving its reliability and effectiveness in the detection of PGs.

## Data Availability

The datasets generated and/or analyzed during the current study are available from the corresponding author upon reasonable request. Any supplementary information and additional raw data supporting the conclusions of this paper will be made available to qualified researchers without undue reservation. However, due to privacy restrictions and data protection regulations, certain datasets (e.g., raw patient data) cannot be made publicly available. Researchers interested in accessing these datasets are encouraged to contact the corresponding author.
